# Powder Metallurgy: Materials and Processing

**DOI:** 10.3390/ma16134575

**Published:** 2023-06-25

**Authors:** Dina V. Dudina, Arina V. Ukhina

**Affiliations:** 1 Lavrentyev Institute of Hydrodynamics, Siberian Branch of the Russian Academy of Sciences, Lavrentyev Ave. 15, Novosibirsk 630090, Russia; 2 Institute of Solid State Chemistry and Mechanochemistry, Siberian Branch of the Russian Academy of Sciences, Kutateladze Str. 18, Novosibirsk 630090, Russia; auhina181@gmail.com

This Special Issue (SI) of *Materials*, “Powder Metallurgy: Materials and Processing”, focuses on the fundamental and applied aspects of materials fabrication by powder metallurgy.

Powder metallurgy is a constantly and rapidly developing field. With the availability of modern powder consolidation technologies and advanced analytical tools, investigations of the material formation mechanisms during sintering under different conditions become possible. Using the obtained knowledge, novel materials are designed in laboratories and produced at the industrial scale. The topics covered in this SI include:

(1) Selection of sintering parameters for achieving desired microstructures and properties;
(2) Microstructure formation mechanisms in sintered materials, including reactively sintered materials;
(3) The influence of the powder morphology and microstructure on their sintering behavior;
(4) Characterization of interfaces in sintered materials;
(5) Sintered metals, alloys, and composites with attractive mechanical and thermophysical properties;
(6) Structural and functional materials produced by powder metallurgy—new approaches to microstructure control;
(7) Advanced sintering methods, including spark plasma sintering;
(8) Comparative investigations of materials obtained via different powder sintering/consolidation methods.

The SI consists of 16 papers (published between 2022 and 2023): a review article [1], a viewpoint paper [2], a communication [3], and thirteen research articles [4,5,6,7,8,9,10,11,12,13,14,15,16]. The papers reflect the current trends in the field: the search for new compositions (not investigated previously) of materials and the development of new processing approaches enabling better control and flexibility of the materials’ microstructures, shapes, and architectures. In the papers published in this SI, materials with traditional and newly elaborated compositions are considered.

The review article [1] is devoted to the microstructure and properties of metal matrix composites reinforced with particles of metallic glass. Sintering of powder mixtures containing a glassy component within the supercooled liquid region of the glass promotes densification and allows preservation of the glassy state in the consolidated composites.

A recent trend in the development of metal matrix composites, strengthening by core–shell particles, is discussed in [2]. During sintering, core–shell morphologies usually form upon the interaction of the added particles with the matrix.

The research papers report recent findings in metallic [4,5,6,7,8,9], ceramic [10,11,12], polymer [3], and composite materials [3,13,14,15,16].

The formation of materials by cyclic impact compaction of an ultra-high-molecular-weight polyethylene without additives and with the addition of nanoscale detonation carbon is reported by Shtertser et al. [3].

The preparation of powders of metallic alloys has been addressed by Petrov et al. [7] and Tikhov et al. [9]. The fabrication of high-entropy alloys via powder metallurgy methods has been presented by Moser et al. [5] and Batraev et al. [6]. The problem of producing parts from 17-4 PH stainless steel by pressing and sintering has been addressed by Kazior [4]. Noteworthy is the increasing attention to the possibilities of field-assisted sintering for the production of metallic alloys and composites, as seen in articles [5,8,14].

Interesting ceramic systems in terms of composition have been investigated in [10,11,12]. Bannykh et al. [10] conducted a systematic study of the products of interaction between the W_2_B and iridium powders. Chorney et al. [11] reported the sintering behavior of Nb_2_O_5_ and Ta_2_O_5_ mixtures and the formation of a ternary oxide. Akopdzhanyan et al. [12] elaborated the synthesis of MgAlON powders via combustion reactions.

Significant attention is given to composites with novel compositions. Guzmán et al. [13] presented the synthesis of Ag-SnO_2_-ZnO via powder metallurgy (ball milling and hot pressing) and the electrical contact behavior of the composites. The developed materials are thought to be able to replace non-environmentally friendly Ag-CdO. Agureev et al. [14] presented a study of the formation of composites in the NiAl-13Cr-13Mo system with small additions of nanoparticles (ZrO_2_, MgAl_2_O_4_). Kozub et al. [15] demonstrated that the addition of a graphite nanopowder to a steel power produces a lubrication effect and provides an improvement in the mechanical properties of the sintered material. Song et al. [16] reported the formation of in situ reinforced Ti6Al4V/TiB composites manufactured using a newly developed approach, namely, hydrogen-assisted sintering of blends containing TiH_2_ and ball-milled Ti + TiB_2_.

We sincerely thank the authors for their contributions and the academic editors and reviewers for their prompt responses and valuable comments.

We extend our gratitude to the *Materials* Editorial Office for providing assistance and technical support at all stages of the manuscript processing.

Volume II of this SI has recently been opened and new submissions are currently welcome. We hope for a continued collaboration.
